# Supplementary material for: Pathological Relevance of Post-Translationally Modified Alpha-Synuclein (pSer87, pSer129, nTyr39) in Idiopathic Parkinson’s Disease and Multiple System Atrophy
Source: Cells. 2022 Mar 6;11(5):906. doi: 10.3390/cells11050906 (PMC8909017; doi:10.3390/cells11050906)
Supplement: Supplementary file 1 [file cells-11-00906-s001.zip › 1. Supplementary Figures S1 and S2 final.pdf]

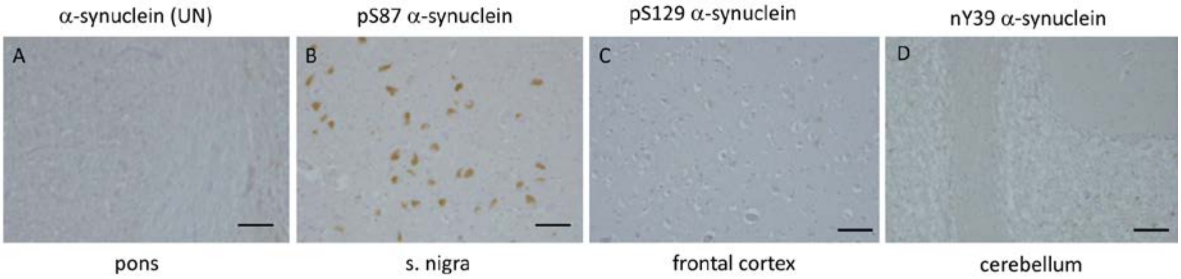

Figure S1. Representative immunohistochemistry illustrations of neurologically normal control cases showing no abnormal α-synuclein deposits when treated with various α-synuclein antibodies. Scale bar 50μm in all.

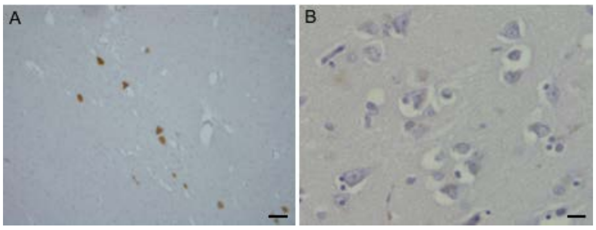

Figure S2. Immunohistochemistry of brain sections from IPD cases showing no appreciable staining within PD nigra (A) or frontal cortex (B) when primary antibodies were omitted but treated with anti-rabbit and anti-mouse secondaries respectively. Scale bar 60μm in A, 15μm in B.
