# Supplementary material for: Pathological Relevance of Post-Translationally Modified Alpha-Synuclein (pSer87, pSer129, nTyr39) in Idiopathic Parkinson’s Disease and Multiple System Atrophy
Source: Cells. 2022 Mar 6;11(5):906. doi: 10.3390/cells11050906 (PMC8909017; doi:10.3390/cells11050906)
Supplement: Supplementary file 1 [file cells-11-00906-s001.zip › 2. Figure S3. Bland-Altman final .pdf]

Supplementary Figure S3

Bland-Altman analysis : LB scores

Bias	9.657
SD of bias	21.48
95% Limits of Agreement	
From	-32.45
To	51.76

A

%Difference vs. average: Bland-Altman of LB Scores

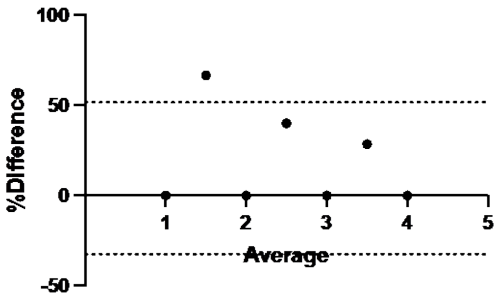

Bland Altman analysis: LN Scores

Bias	7.059
SD of bias	25.89
95% Limits of Agreement	
From	-43.70
To	57.81

B

%Difference vs. average: Bland-Altman of LN Scores

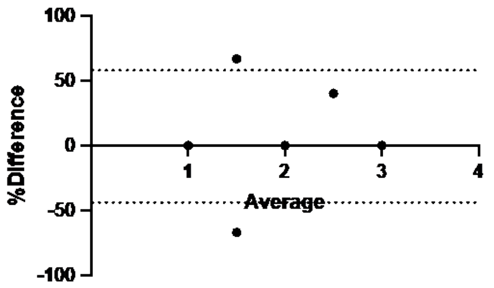

Figure S3: Intra-rater (one scorer blind to status of cases) analysis of Bland Altman analysis of IHC scoring: A) lewy body scores; B) LN scores. There was~84% agreement for LBs and ~86% agreement for LN scores.
