# Supplementary material for: Pathological Relevance of Post-Translationally Modified Alpha-Synuclein (pSer87, pSer129, nTyr39) in Idiopathic Parkinson’s Disease and Multiple System Atrophy
Source: Cells. 2022 Mar 6;11(5):906. doi: 10.3390/cells11050906 (PMC8909017; doi:10.3390/cells11050906)
Supplement: Supplementary file 1 [file cells-11-00906-s001.zip › 3. Table S1 final.pptx]

## Slide 1
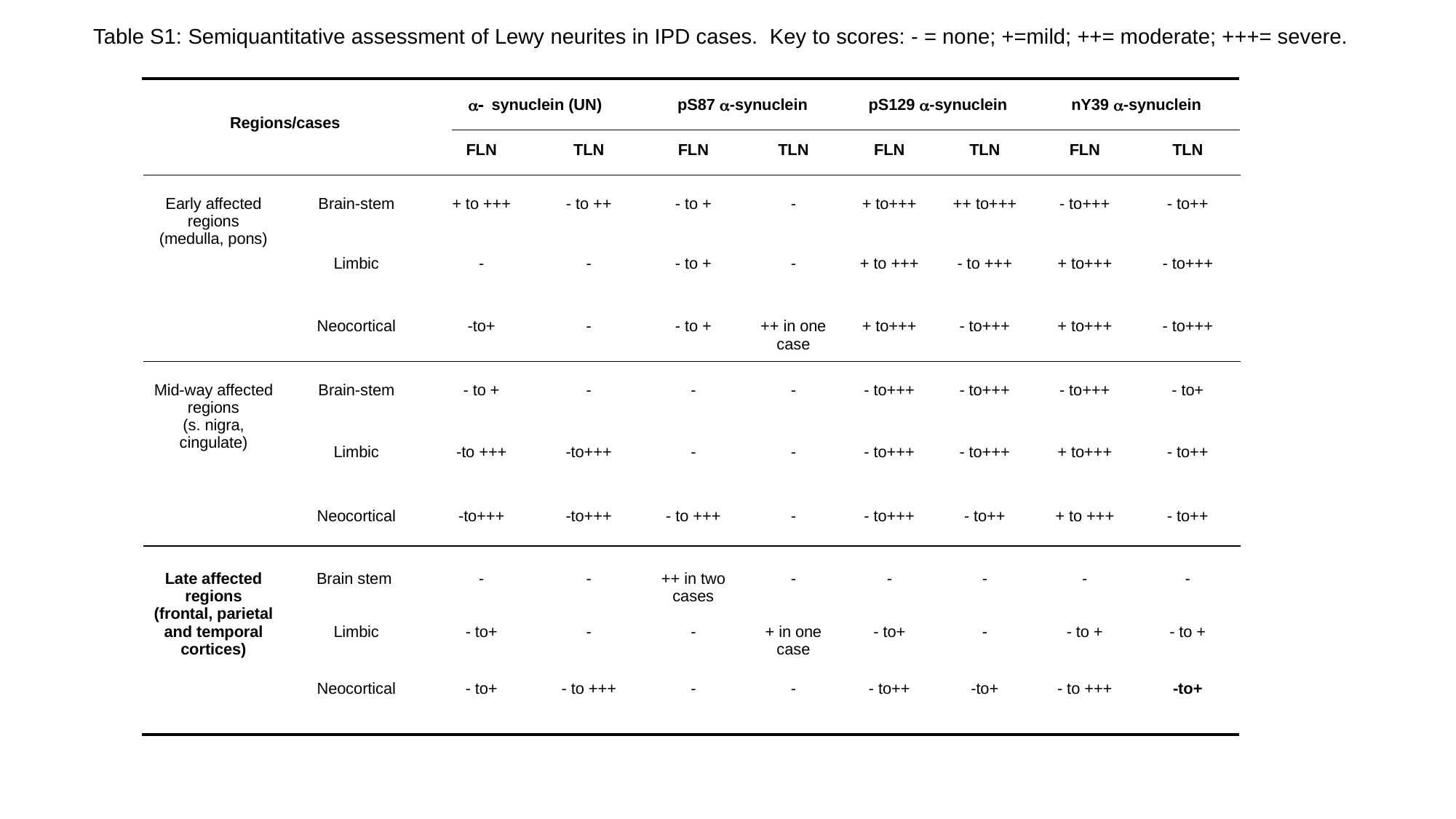

Table S1: Semiquantitative assessment of Lewy neurites in IPD cases. Key to scores: - = none; +=mild; ++= moderate; +++= severe.
| Regions/cases | | a- synuclein (UN) | | pS87 a-synuclein | | pS129 a-synuclein | | nY39 a-synuclein | |
| --- | --- | --- | --- | --- | --- | --- | --- | --- | --- |
| | | FLN | TLN | FLN | TLN | FLN | TLN | FLN | TLN |
| Early affected regions (medulla, pons) | Brain-stem | + to +++ | - to ++ | - to + | - | + to+++ | ++ to+++ | - to+++ | - to++ |
| | Limbic | - | - | - to + | - | + to +++ | - to +++ | + to+++ | - to+++ |
| | Neocortical | -to+ | - | - to + | ++ in one case | + to+++ | - to+++ | + to+++ | - to+++ |
| Mid-way affected regions (s. nigra, cingulate) | Brain-stem | - to + | - | - | - | - to+++ | - to+++ | - to+++ | - to+ |
| | Limbic | -to +++ | -to+++ | - | - | - to+++ | - to+++ | + to+++ | - to++ |
| | Neocortical | -to+++ | -to+++ | - to +++ | - | - to+++ | - to++ | + to +++ | - to++ |
| Late affected regions (frontal, parietal and temporal cortices) | Brain stem | - | - | ++ in two cases | - | - | - | - | - |
| | Limbic | - to+ | - | - | + in one case | - to+ | - | - to + | - to + |
| | Neocortical | - to+ | - to +++ | - | - | - to++ | -to+ | - to +++ | -to+ |
